# Gordie Howe’s “Miraculous Treatment”: Case Study of Twitter Users’ Reactions to a Sport Celebrity’s Stem Cell Treatment

**DOI:** 10.2196/publichealth.5264

**Published:** 2016-03-09

**Authors:** Li Du, Christen Rachul, Zhaochen Guo, Timothy Caulfield

**Affiliations:** ^1^ Health Law Institute Faculty of Law University of Alberta Edmonton, AB Canada; ^2^ School of Linguistics and Language Studies Carleton University Ottawa, ON Canada; ^3^ Department of Computing Science University of Alberta Edmonton, AB Canada; ^4^ Health Law Institute, Law Centre, University of Alberta Edmonton, AB Canada

**Keywords:** Gordie Howe, stem cell treatment, stem cell tourism, social network, Twitter, infodemiology, infoveillance

## Abstract

**Background:**

Former Detroit Red Wing Gordie Howe received stem cell (SC) treatment in Mexico in December 2014 for a stroke he suffered in October 2014. The news about his positive response to the SC treatment prompted discussion on social networks like Twitter.

**Objective:**

This study aims to provide information about discussions that took place on Twitter regarding Howe’s SC treatment and SC treatment in general. In particular, this study examines whether tweets portrayed a positive or negative attitude towards Howe’s SC treatment, whether or not tweets mention that the treatment is unproven, and whether the tweets mention risks associated with the SC treatment.

**Methods:**

This is an infodemiology study, harnessing big data published on the Internet for public health research and analysis of public engagement. A corpus of 2783 tweets about Howe’s SC treatment was compiled using a program that collected English-language tweets from December 19, 2014 at 00:00 to February 7, 2015 at 00:00. A content analysis of the corpus was conducted using a coding framework developed through a two-stage process.

**Results:**

78.87% (2195/2783) of tweets mentioned improvements to Howe’s health. Only one tweet explicitly mentioned that Howe’s SC treatment was unproven, and 3 tweets warned that direct-to-consumer SC treatments lacked scientific evidence. In addition, 10.31% (287/2783) of tweets mentioned challenges with SC treatment that have been raised by scientists and researchers, and 3.70% (103/2783) of tweets either defined Howe as a “stem cell tourist” or claimed that his treatment was part of “stem cell tourism”. In general, 71.79% (1998/2783) of tweets portrayed a positive attitude towards Howe’s SC treatment.

**Conclusions:**

Our study found the responses to Howe’s treatment on Twitter to be overwhelmingly positive. There was far less attention paid to the lack of scientific evidence regarding the efficacy of the treatment. Unbalanced and uncritical discussion on Twitter regarding SC treatments is another example of inaccurate representations of SC treatments that may create unrealistic expectations that will facilitate the market for unproven stem cell therapies.

## Introduction

Over the past few decades, stem cell (SC) research has gained considerable attention from the international bioscience community. With continued developments in basic SC research, the potential for clinical application of SCs has generated a great deal of hope for therapies and treatments for a wide range of diseases, including neurodegenerative diseases and fatal illnesses that cannot be treated with existing medical treatments [1,2]. To date, very few SC therapies have received approval from governmental regulatory authorities in countries like the United States and Canada [3]. Despite this, many direct-to-consumer clinics exist in countries throughout the world that offer unproven SC therapies (eg, United States, China, and Ukraine) [4-8]. Research has suggested that many patients, often with serious and/or terminal conditions, travel to these clinics to receive SC therapies, a phenomenon that is often referred to, controversially, as “stem cell tourism” [9-12].

Stories about well-known individuals, such as athletes, seeking unproven SC treatments to heal injuries or accelerate the speed of recovery, have proliferated in recent years [13,14]. One study, for instance revealed that between 2012 and 2013 numerous National Football League (NFL) players claimed to have received SC therapies in the United States or abroad (eg, Germany and South Korea) [14]. A more recent high-profile example is that of Gordie Howe, a former Detroit Red Wing hockey player nicknamed “Mr. Hockey”. In December 2014, Howe traveled outside the United States to receive SC treatment in Mexico for a stroke he suffered on October 26, 2014 [15]. The news release about Howe’s positive response to the SC treatment attracted a great amount of attention in the news and sports media, and on social networks. A study of news stories and readers’ comments about Howe’s SC treatment revealed that the efficacy of Howe’s treatment is often assumed and that public debates tend to focus on the lack of access to SC treatments more than the lack of scientific evidence and possible risks associated with the unproven therapies [16].

Popular media are an important source of health information for the public and can both shape and reflect public opinion of major health stories [17-21]. Media coverage has also been shown to play a role in creating public interest in new biomedical technologies [6,22,23], and influencing health policy debates [24]. More recently, social networking websites that facilitate online interaction and communication, like Twitter, have allowed for rapid and widespread dissemination of important health information [25-27]. Twitter users can post, share or re-tweet messages with a 140-word limit to express their opinions and join the public debate. With more than 300 million users, Twitter offers a rich source of online discussion and debate between users that include individual members of the public and organizations from news media to academic institutions. As such, it provides a rich source naturalistic data to gauge health trends and public responses to major health issues [28,29]. This approach is also known as infodemiology or infoveillance [29]. In addition, celebrities’ engagement with the public through the popular media, as well as the information shared on social networking sites, can influence individual health-related attitudes and behaviors [30,31]. Research using Twitter may be limited because users include both organizations, who may have vested interests, and individuals, commonly college-educated adults under the age of 50 [25], but it can still provide valuable insights into what kind of information the public are exposed to and interact with.

Although popular media, including Twitter, have been shown to contribute to the hype surrounding stem cell research [16,32-33], less is known about whether discussions on Twitter about a specific individual’s SC treatment also contributes to this hype. Howe’s SC treatment and its concomitant Twitter discussions provide the opportunity for exploring this topic, specifically discussions about an individual’s SC treatment on Twitter when such treatments are associated with a sports celebrity. This study consists of a content analysis of tweets about Howe’s SC treatment that were posted within five weeks after news of his treatment was announced, and examines the information provided in and the tone of tweets about Howe’s SC treatment in Mexico.

## Methods

A corpus of tweets was compiled by developing a program using Python programming language through Topsy API that allowed for the collection of tweets that contain specific terms within a specified time frame [34]. We used the Topsy API program because it is Twitter’s only certified partner and it provides unlimited access to and full coverage of tweets since Twitter was launched in 2006. Our program was developed to automatically crawl English-language tweets containing the search terms “Gordie Howe” and “stem cell” that were posted on Twitter from December 19, 2014 at 00:00, the date that the story first appeared in the media, to February 7, 2015 at 00:00, the time when we started data collection. We limited our search terms to compile a manageable sample of tweets. As such, our corpus may not contain related tweets with variations on the terms, but still represents a large sample that provides insight into the nature of the discussions about Gordie Howe’s stem cell treatment in Mexico. The program collected 2788 tweets that included the date and time, tweet content, tweet URL, its author, and author URL. We excluded 5 tweets that were not directly and specifically related to Howe’s SC treatment story, resulting in a final data set of 2783 tweets.

A content analysis of the tweets was conducted in two stages. First, we conducted an exploratory thematic analysis of a random sample of 10% of tweets from the data set by which we established a set of common themes (eg, improvements of Howe’s condition, risks associated with Howe’s treatment) that appeared in the representations of Howe’s SC treatment story on Twitter. Second, we developed a coding framework that was based on the common themes that emerged during the exploratory thematic analysis. The coding framework was also informed by previous coding frameworks developed by our team [35-37]. The coding framework included 7 items about whether tweets (1) included a claim that Howe’s health condition improved; (2) mentioned that Howe’s SC treatment is unproven; (3) defined the treatment as a miracle or miraculous; (4) mentioned or defined Howe’s treatment as part of SC tourism; (5) mentioned challenges raised by scientists, researchers, and/or ethicists; and (6) mentioned risks associated with Howe’s SC intervention. Finally, the coding framework included an item that considered the overall tone, which assessed whether the attitude conveyed in a tweet towards Howe’s SC treatment was positive, negative or neutral.

Due to the large data set, a single coder with expertise in stem cell tourism applied the coding framework to the entire data set. In order to minimize coder bias for subjective items in the coding framework, an independent coder without expertise applied the same coding framework to 10% of the data set to assess inter-coder reliability. Inter-coder reliability was calculated using Cohen’s kappa (K), which generated K scores on different coding categories in the range of 0.750-1.000, thereby indicating substantial or almost perfect agreement as based on the Landis & Koch’s benchmark for interpreting kappa [38].

## Results

In the first five weeks following the announcement about Howe’s SC treatment, much of the discussion on Twitter appears to have been prompted by the publication of news media reports. A press release announcing Howe’s SC treatment and recovery was published on December 19, 2014, and was picked up by several news media outlets that same day [16,39-42]. Over 710 tweets from our corpus were posted on that day. Following the initial interest, another spike in published tweets in our corpus (419 tweets) appeared on January 27, 2015 in response to the CBC Sports report that Howe showed dramatic improvement after his SC treatment. Tweets that generated re-tweets or were shared were often article titles published in media sources like newspapers and sports websites, with fewer original tweets coming from individuals (Tables 1 and 2).

**Table 1:** Examples of the most frequently re-tweeted messages that show positive attitude towards Howe’s SC treatment.

Tweets	Original sender	Number of times re-tweeted or shared^a^
Gordie Howe makes “amazing” recovery following stem cell treatment in Mexico	NBC Sports	188
Gordie Howe shows improvement after stem cell treatment	CBC Sports	157
VIDEO: Stroke victim Gordie Howe, now (back) playing hockey! Stem Cell Co. CEO joins me (ClinicalTrials website)	Keith Olbermann	68
Gordie Howe continues progress following stem cell treatment	Michigan Live	58
Experimental stem cell treatment key in Gordie Howe’s dramatic improvement	The Globe and Mail	55
Gordie Howe shows dramatic improvement after stem cell treatment	CBC Sports	54
Gordie Howe back stick-handling after stem cell treatment	The Detroit News	51
Gordie Howe's “miraculous” recovery after stroke credited to stem cell treatments in Mexico, says family.	SportsNET Canada (online)	46

^a^Re-tweets are defined as tweets started with the “RT”; shared tweets are those tweets posted without the “RT” but having exactly the same tweet content.

**Table 2:** Examples of tweets that mentioned challenges raised by scientists and/or researchers regarding Gordie Howe’s stem cell treatment.

Tweets	Original senders	Number of times re-tweeted or shared^a^
Gordie Howe, stem-cell tourist: experts warn of a worrisome trend	Ottawa Citizen	79
Gordie Howe's stem cell therapy raises concerns among regenerative medicine	National Post	76
Gordie Howe's stem cell therapy raises concerns among medical experts	CTV News	40
Gordie Howe’s “miracle” in Mexico stirs experts’ doubts about stem-cell therapy	The Global and Mail	30

^a^Re-tweets are defined as tweets started with the “RT”; shared tweets are whose tweets posted without the “RT” but having exactly the same tweet content.

The majority of tweets in our corpus cited that Howe’s health improved following his SC treatment in Mexico (78.87%, 2195/2783). Many of these tweets used descriptive words, such as “miraculous”, “dramatic”, “amazing”, and “remarkable”, to describe the degree to which Howe’s condition had improved (Table 1). In addition, detailed descriptions of the hockey player’s progress were frequently re-tweeted, such as “Gordie Howe goes from not being able to walk to pushing a cart around following stem cell treatment” and “Gordie Howe back stick-handling after stem-cell treatment” (Table 1). Other common topics in the tweets included excerpts from interviews with Howe’s family members that confirmed and provided evidence for the improvements in Howe’s health, for example, “Gordie Howe's family said Gordie has made a miraculous recovery with stem cell injections” and “Mark Howe says Gordie's health has improved dramatically since stem cell treatment” (Tables 1 and 2).

Tweets with criticisms or that raised concerns were less frequent. Of the 2783 tweets in the corpus, only one tweet mentioned that Howe’s SC treatment was unproven (which was posted by one of this paper’s authors); and 3 tweets warned that SC treatments lacked scientific evidence and that further research was needed to determine the efficacy and safety of SC treatments. While 10.31% of tweets (287/2783) mentioned scientists and researchers have cited challenges associated with Howe’s SC treatment, only 5 tweets directly considered its potential health risks (Table 2). References to stem cell tourism also appeared in tweets, with 3.70% of tweets (103/2783) either defining Howe as a “stem cell tourist” or describing his treatment in Mexico as “stem cell tourism”. Among this 3.70% (103/2783), the most frequently re-tweeted message was: “Gordie Howe, stem-cell tourist: experts warn of worrisome trend”, which was re-tweeted 79 times (Table 2).

Overall, Howe’s SC treatment was represented as a success story. The majority of tweets were positive (71.79%, 1998/2783). These tweets either used positive words to describe the treatment or provided details to remark on the great improvement in Howe’s health. In contrast, only 14.73% (410/2783) of tweets were negative in tone, and usually focused on criticisms of Howe’s decision to travel outside of the United States for treatment and concerns that medical experts have with the treatment. Some of the tweets (13.47%, 375/2783) were considered neutral since they simply relayed news of Howe’s treatment (eg, “Gordie Howe underwent stem cell clinical trial in Mexico” and “Marty Howe recounts the trip to Mexico for Gordie Howe's stem cell treatment”).

## Discussion

### Principal Findings

Our data show an overwhelmingly positive attitude towards Howe’s SC treatment, about 71.79% (1998/2783) of our corpus. In comparison, safety concerns and potential harms associated with unproven SC treatments (eg, physical harm, financial exploitation, and creation of unrealistic expectations) were rarely mentioned. These results may suggest misunderstandings of the current state of SC research. Previous studies have found news media representations of SC research is often inappropriately “hyped”. For example, studies have found that the coverage of SC research often provides overly optimistic estimates of the length of time it takes for research to reach the clinics [33,43]. Given that many of the original tweets come from news media sources like newspapers and sports websites, our results illustrate how social networks, such as Twitter, bias exposure to information and contribute to the dissemination of these overly optimistic portrayals [16,26,32,33,44].

Previous studies have explored the nature and role of social media in the context of stem cells and found representations to be, in general, predominantly positive in tone [32,33]. Our results fit with this trend. We found that a large number of tweets were published immediately following the initial press release and media reports detailing Howe’s treatment and recovery. The immediacy of reactions on Twitter highlights the power of celebrity to generate public interest. This finding is consistent with other research on celebrities’ impact on the public regarding health-related issues [31,45,46]. For example, Angelina Jolie’s announcement of her genetic predisposition to cancer and her decision to have prophylactic surgeries [47,48], resulted in an increase in interest in breast cancer genetic testing and prophylactic surgery [49].

Since Howe received his SC treatment outside the United States, issues surrounding stem cell tourism were raised on Twitter. However, compared to tweets focused on the improvements in Howe’s health, the number of tweets that critiqued the phenomenon of stem cell tourism accounted for a very small portion (3.70%, 103/2783). We also observed that news media (eg, National Post and The Globe and Mail), and academic groups (eg, medical experts, scientists, and ethics scholars) did provide more critical views on, and warnings about, the implications of Howe’s SC treatment. For example, the National Post stated: “Gordie Howe's stem cell therapy raises concerns among regenerative medicine”, while The Globe and Mail stated: “Gordie Howe’s ‘miracle’ in Mexico stirs experts’ doubts about stem-cell therapy” [50,51]. Unfortunately, these more circumspect voices were eclipsed by the positive reactions in the majority of tweets and, as such, the social media coverage was unbalanced.

### Limitations

Our study had several limitations. The search terms we used to collect tweets were limited and related tweets with other variations on the search terms may have been excluded. The corpus was analyzed by only a single coder with expertise in stem cell tourism, whose perspective may have influenced results, but we have taken steps to assess the reliability of the coding. Our data analysis did not include Web links included in tweets, so we are unable to evaluate the spectrum of information sources reached through social media, or make claims regarding which types of news media are most frequently shared and may have more social impact. We also did not collect background information on Twitter users. Therefore, we cannot evaluate other contextual factors, such as whether tweets about Gordie Howe’s SC treatment originated from a specific country. Further research is needed to examine to what extent Twitter has helped to increase the public’s scientific understandings of SC research and treatment.

### Conclusions

Much of the Twitter discussion about Howe’s SC treatment was prompted by news media reports. Our research highlights how tweets expressed a largely positive attitude toward Howe’s SC treatment. There was little discussion about the lack of scientific evidence on the efficacy of SC treatments. Less attention was also paid to the potential risks and safety concerns associated with unproven SC treatments. Given these findings, it seems reasonable to conclude that discussions on Twitter regarding celebrities’ SC treatments may contribute to the hype around SC research and to the dissemination of inaccurate representations of the benefits and risks associated with unproven treatments. This may, in turn, mislead patients and the public and prompt their engagement with clinics that market unproven stem cell products and procedures [52].

## Abbreviations

SC: stem cell
